# Trapped In Transit – A Case Report of a Pediatric Gastric Bezoar Causing Intermittent Small Bowel Obstruction

**DOI:** 10.5070/M5.52259

**Published:** 2026-04-30

**Authors:** Jonah A Frueh, Arinzechukwu M Obegolu, Anil G Rao, Amanda E Mulcrone

**Affiliations:** * Advocate Christ Medical Center, Department of Emergency Medicine, Oak Lawn, IL; ^ Advocate Christ Medical Center, Department of Pediatrics, Oak Lawn, IL; † Advocate Christ Medical Center, Department of Pediatric Radiology, Park Ridge, IL; ** Advocate Christ Medical Center, Department of Pediatric Emergency Medicine, Park Ridge, IL

## Abstract

A 14-year-old female patient presented to the pediatric emergency department (ED) with chief complaints of abdominal pain, vomiting, and unintentional weight loss. A palpable abdominal mass was noted on exam, prompting a computed tomography (CT) scan which revealed a gastric bezoar that had fragmented, leading to intermittent and spontaneously resolving small bowel obstruction. The patient underwent surgical removal of the bezoar for definitive management.

This report highlights a novel case of spontaneous bezoar fragmentation and its associated imaging findings. Because this phenomenon represents the description of a rare occurrence, the goal is to enhance the emergency medicine providers’ understanding of an uncommon cause of abdominal masses and small bowel obstruction (SBO).

**Topics:**

Pediatrics, bezoar, bowel obstruction.

**Figure:**
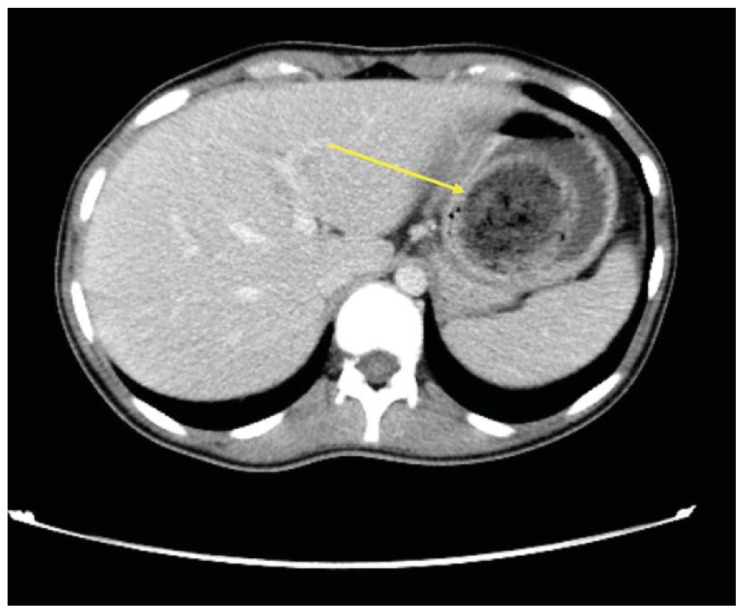


**Figure:**
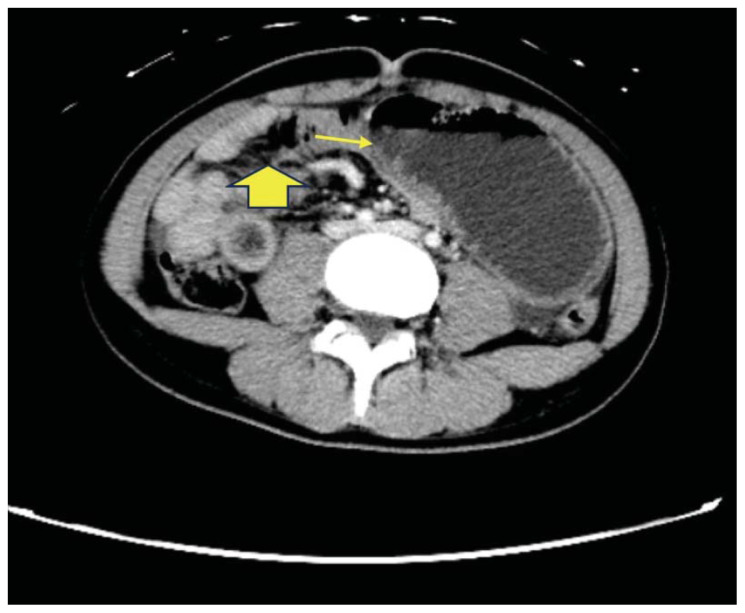


**Figure:**
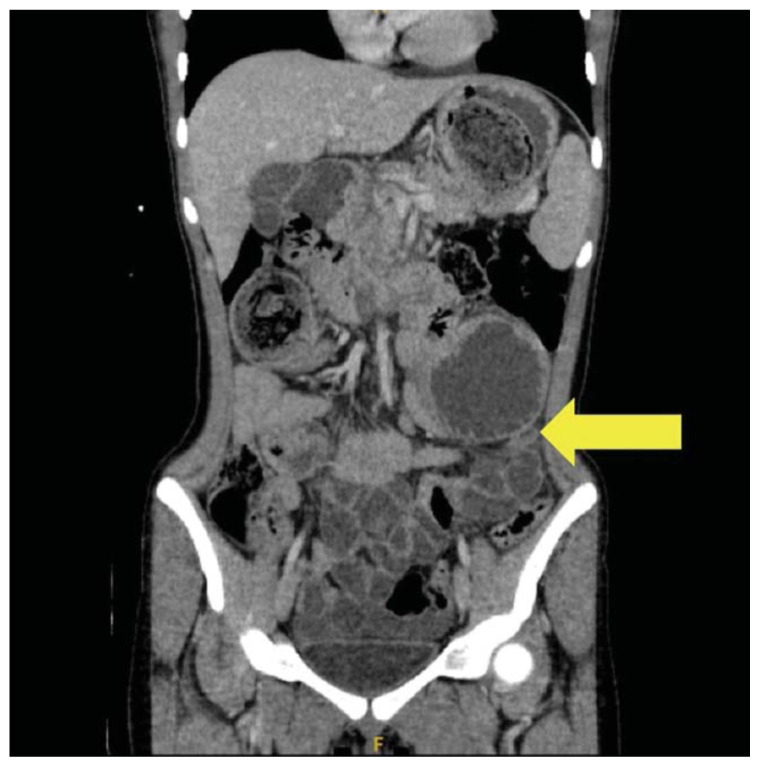


**Figure:**
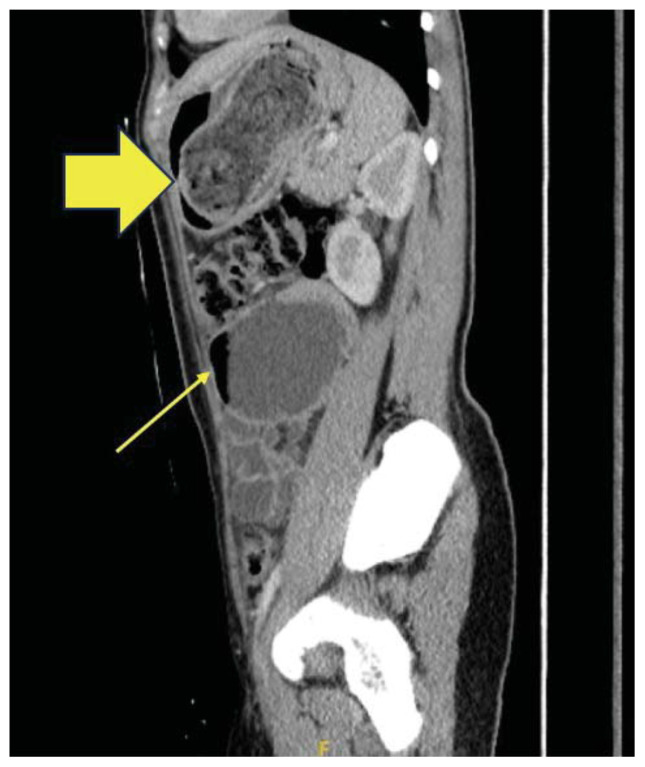


**Figure:**
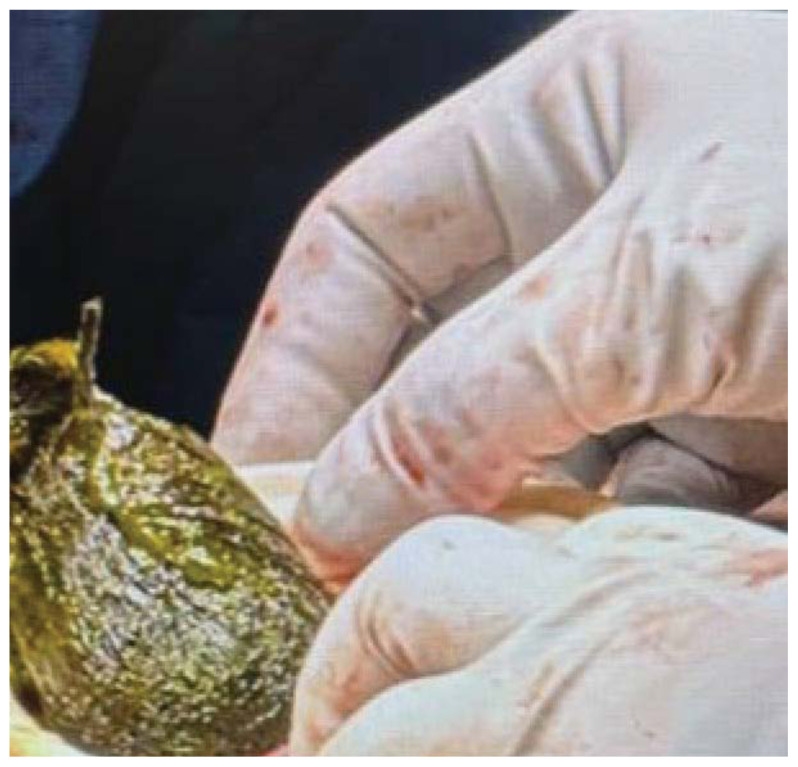


**Figure:**
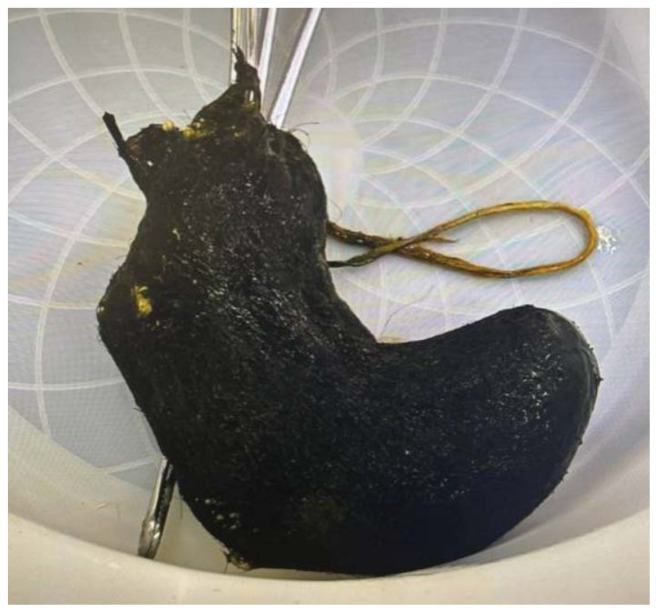


## Brief introduction

Gastric bezoars are indigestible materials that collect and accumulate in the gastrointestinal (GI) tract.^1^–^7^ They are typically classified based on their composition. Hair-containing bezoars are referred to as trichobezoars and predominantly affect female patients.^1^–^3^,^6^,^8^,^9^ Bezoars have been known to cause nausea and vomiting and severe complications such as bowel obstruction, ulceration, perforation and pancreatitis.^1^–^7^ Bezoars may also present clinically as palpable abdominal masses.^6^,^7^ They often require procedural removal due to possibility of severe complications and can be retrieved by multiple techniques, including endoscopy, laparotomy and laparoscopy.^1^–^7^ They are rare overall, with an estimated prevalence of as low as 0.068%.^10^

The differential diagnosis for palpable abdominal masses is broad. It can include splenomegaly, hepatomegaly, malignant or benign neoplasms of intra-abdominal organs, malformation, or disorganized development of the gastrointestinal (GI) tract, hernias, lipomas, hematomas or GI obstructions, such as in our patient’s case. This report presents an interesting case of a trichobezoar causing intermittent SBO due to spontaneous fragmentation.

## Presenting concerns and clinical findings

A 14-year-old girl with history of anxiety, depression, attention deficit hyperactivity disorder and post-traumatic stress disorder initially presented to the emergency department (ED), with intermittent, sharp epigastric abdominal pain that had persisted for 10 days. She reported a transient epigastric “knot” that she could feel, which would intermittently and spontaneously resolve. She denied any fever or diarrhea. She had a history of constipation, with bowel movements occurring every 2–3 days. On initial exam, no abdominal mass was noted, and the patient corroborated that she didn’t feel the “knot” at time of ED evaluation. A urinalysis and urine pregnancy test were obtained and were unremarkable. Diagnostic considerations included a hernia of the abdominal muscular wall or constipation. She was discharged with return precautions and a bowel regimen for presumed constipation.

One month later, the patient returned to the ED with persistent epigastric abdominal pain, now accompanied by bilious emesis. She reported a relief of the pain in a knees-to-chest or left lateral decubitus position. The patient had attempted the previously recommended bowel regimen with no relief. She also endorsed an unintentional 16-pound weight loss in the past 5 months.

On physical exam, the patient was noted to have normal vital signs. The patient was notably uncomfortable laying on her left side. She had generalized abdominal tenderness with guarding and a firm, palpable mass in the left upper quadrant and epigastrium along the inferior left ribcage. Delayed capillary refill time as well as dry mucous membranes were also noted. The patient appeared pale. Due to these physical exam findings and overall concern about the patient’s palpable abdominal mass on exam, a CT scan was ordered for further evaluation, with concern for the mass as the etiology of the patient’s presenting symptoms of pain and weight loss. Laboratory studies including complete blood count (CBC), comprehensive metabolic panel (CMP), lipase, and urinalysis were ordered as well.

## Significant findings

The CT scan identified a heterogenous, mass-like lesion extending from the gastric fundus to the antrum, along with a small bowel obstruction (SBO) and significant small bowel dilation. An additional smaller heterogenous mass-like lesion was seen in the small bowel, causing the SBO. Given the masslike lesion’s mixed attenuation and presence of internal air, a gastric bezoar was strongly suspected.

A large portion of the bezoar was seen in the gastric fundus, identified by a yellow arrow on axial CT. Note the heterogeneous density overall with a hypodense, well circumscribed “encapsulating wall,” which is unique to bezoars compared to stool.^14^ Also seen on axial CT imaging was a dilated loop of small bowel with a gas-fluid level distal to the bezoar, showing evidence of obstruction and identified by a small yellow arrow. The large yellow arrow on this same image points to the bezoar in the proximal small bowel. A dilated loop of small bowel was also identified in the left upper quadrant on a sagittal CT image, identified by a small yellow arrow. The large gastric bezoar is also seen in the stomach on this same sagittal image, marked by the large yellow arrow. The lead point of the SBO, recognized by dilated bowel proximal to decompressed bowel, can be seen on a coronal CT image with a yellow arrow pointing to this finding.

## Patient course

With concern for gastric bezoar, the ED physicians obtained additional history from the patient. She revealed that, due to her anxiety, she had previously ingested her hair, pieces of cloth from t-shirts and blankets, “clay from the wall,” and pages of books. The patient noted that she had currently stopped ingesting these nonfood items, reporting the last occurrence of this approximately a year ago. With confirmation of the ingestion of indigestible materials, the radiologist felt confident in diagnosing a gastric bezoar with spontaneous fragmentation, explaining the multiple lesion locations on imaging and the intermittent nature of the patient’s symptoms.

The patient’s CBC revealed a hemoglobin of 5.7 g/dL (normal range 12.0 – 15.5 g/dL), indicating anemia. Clinically, the patient was noted to be stable. Hematology was consulted and recommended the patient receive intravenous iron in the ED, rather than blood products. The remainder of laboratory studies were unremarkable.

The pediatric surgery team was consulted from the ED, and the patient was admitted for surgical management. The complete bezoar was removed from the gastric compartment intraoperatively. Images from operative removal are included.

The surgical procedure began laparoscopically, but due to significant gastric distension and dilated small bowel loops, it was converted to an open laparotomy. Intraoperatively, a loop of jejunum was found partially fused to itself with an inflammatory rind. Upon minimal probing, a significant jejunal perforation was identified, attempting to seal off itself off with the inflammatory rind. A 15 cm segment of affected surrounding jejunum was resected. Distal to this, a jejunal obstruction caused by a bezoar was noted, with the proximal bowel appearing dilated and edematous. The bezoar was removed, and approximately 3 cm of unhealthy bowel was resected in this area. A gastrotomy was then performed on the stomach near the greater curvature, and the gastric bezoar was extracted in its entirety before the stomach and abdomen were surgically closed.

Postoperatively, the patient was admitted to the children’s hospital for recovery. She had a nasogastric (NG) tube to low-intermittent suction with bilious output and a wound vacuum in place. Due to the jejunal perforation seen during surgery, she was started on a 5-day course of ceftriaxone and metronidazole.

On post-operative day 3, a fluoroscopic upper GI study demonstrated prompt gastric emptying without obstruction or leak. Her NG tube was removed, and her diet was advanced as tolerated. The wound vacuum was removed on post-op day 4. During her hospitalization, the pediatric hematology team evaluated her anemia and suspected it to be secondary to iron deficiency and pica. She received two iron infusions during her admission because the hematologist was concerned that her recent intestinal surgery would impair oral iron absorption. Hemoglobin levels trended upward, reaching 8.4 g/dL prior to discharge. The patient was discharged after she was able to tolerate a full diet and had completed the 5-day course of antibiotics.

The patient followed up with the surgery team three weeks later in the outpatient clinic and was noted to be doing well. She was cleared for full physical activity with no need for further follow-up. She received a total of three outpatient IV iron infusions with the hematology team before transitioning to oral iron supplementation.

## Discussion

This case reports a rare instance of spontaneous gastric bezoar fragmentation leading to intermittent small bowel obstruction (SBO). Spontaneous fragmentation of a gastric bezoar has been previously characterized in the literature but still represents an extremely rare occurrence.^11^ This case highlights several important considerations in the diagnosis and management of bezoars including the role of different imaging modalities, the potential for conservative treatment, associated psychiatric comorbidities, and the risks of diagnostic anchoring bias.

Computed tomography is one of the more sensitive and specific imaging techniques to diagnose a bezoar. Surgery-confirmed correct identification of bezoar by CT has been reported as high as 97%. 12 It provides clear visualization of characteristic features of a bezoar, such as an intraluminal gas-containing mass that appears encapsulated.^12^–^15^ However, differentiating bezoars from small-bowel feces on CT can be challenging. Specific CT signs, such as a free standing area of encapsulated debris, the same density as fat tissue intra-luminally in the intestine, and the presence of a similar lesion in the stomach, can be helpful in distinguishing bezoars from other intraluminal masses and feces.^12^–^15^

Ultrasound is a useful alternative to CT imaging in pediatric patients due to its lack of radiation exposure, easy accessibility, portable nature, and lower costs. Radiation exposure from CT scans in young children can increase the lifetime risk of a radiation-induced cancer and should be considered.^16^ However, the risk of radiation-induced cancer from exposure to diagnostic imaging is overall very low. The benefits of an abdomen and pelvis CT in the acute abdomen in general outweigh the small theoretical risks of radiation exposure. The American College of Radiology recommends ultrasound as an initial imaging modality for evaluating intra-abdominal masses in children.^17^ For multiple bezoars or gastric lesions, sonography is less sensitive than CT for identification, which can lead to delays in diagnosis and treatment.^12^,^13^ Abdominal radiographs are the least sensitive imaging modality and are often unreliable in detecting bezoars, though they may still show gastric distention.^12^,^13^

While bezoars often require surgical or endoscopic removal, bezoars which resolved spontaneously, without any intervention, have been previously described.^18^ This suggests that in the absence of severe complications such as gastric outlet obstruction or SBO, non-invasive management can be considered.^18^ Endoscopic techniques utilizing laser therapy or electrohydraulic lithotripsy have also been previously used to break down bezoars within the stomach.^19^

Enzymatic therapy, such as with papain, cellulase, and cysteine proteases, are an increasingly popular option for bezoar breakdown, allowing for easier endoscopic removal or spontaneous passage.^20^ However, efficacy and dissolution using enzymatic breakdown may be dependent upon bezoar size and composition.^20^ This approach may therefore be applicable to predominantly small bezoars and in patients without acute symptoms. In our patient’s case, the bezoar led to both gastric and small bowel obstruction, necessitating surgical intervention.

Bezoars, particularly trichobezoars, are frequently associated with psychiatric conditions such as trichotillomania (the compulsive urge to pull out one’s hair), trichophagia (the ingestion of hair), anxiety, and pica (the consumption of nonfood items).^8^,^9^,^21^–^23^ As such, it is important to consider addressing the underlying psychiatric issues in preventing a bezoar recurrence.^21^,^23^

Severe cases of pica have also been reported to contribute to bezoar formation and have led to complications such as intestinal obstruction, perforation, and ischemia.^24^–^26^ The ingested substances in pica are thought to interfere with enteral iron absorption and contribute to iron deficiency anemia.^27^ Individuals with pica may have lower levels of serum ferritin and total body iron compared to those without pica.^28^ Pica may alternatively be the body’s response to being iron deficient.^29^ The consumption of ice is commonly seen in pica, and may be a compensatory behavior to provide relief from oral inflammation or glossitis, symptoms sometimes seen in iron deficiency.^29^

This case also highlights the potential pitfall of diagnostic anchoring, particularly in pediatric patients where constipation is a common diagnosis. Anchoring bias can lead clinicians to delay consideration of alternative causes such as bezoars or other obstructive pathologies, especially given their relative rarity.^30^

Misdiagnoses in children with constipation is unfortunately common.^24^ One study found that children who underwent abdominal radiographs for suspected constipation had higher rates of misdiagnosis, and that severe pathologies, such SBO and constipation were missed in some cases.^31^ In our patient, diagnosis of bezoar causing SBO was further obfuscated by the protracted time course of the patient’s history of bezoar and pica. Having ceased ingesting non-digestible material for over a year, the SBO developed after the bezoar had much time to begin to break down. Such errors and pitfalls could perhaps be minimized by a more detailed history and clinical examination in conjunction with appropriate laboratory and imaging tests.

Weight loss, GI bleeding, persistent fever, chronic severe diarrhea, and significant vomiting are red flag symptoms that should prompt further investigation in pediatric patients with abdominal pain.^32^,^33^ In this case, the patient’s unintentional 16-pound weight loss, intermittent obstructive symptoms, and failure to improve with constipation treatment were all important symptoms noted in the patient’s history which influenced the team’s evaluation and diagnostic work-up.

In conclusion, this case demonstrates the importance of maintaining a broad differential diagnosis for pediatric abdominal pain, particularly when red flag symptoms are present. Though this is a singular case, it illustrates that bezoars should be considered in patients with a history of pica, trichotillomania, psychiatric disorders, or unexplained obstructive symptoms. As previously stated, this patient had multiple visits prior to definitive imagining and diagnosis. Imaging plays a critical role in diagnosis with CT offering the highest sensitivity, though ultrasound remains a useful option in pediatric patients. Appropriate history and clinical evaluation can also help in appropriate management and patient care.

## Supplementary Information




















