# Supplementary figures and images for: Trapped In Transit – A Case Report of a Pediatric Gastric Bezoar Causing Intermittent Small Bowel Obstruction
Source: J Educ Teach Emerg Med. 2026 Apr 30;11(2):V41–7. doi: 10.5070/M5.52259 (PMC13152341; doi:10.5070/M5.52259)

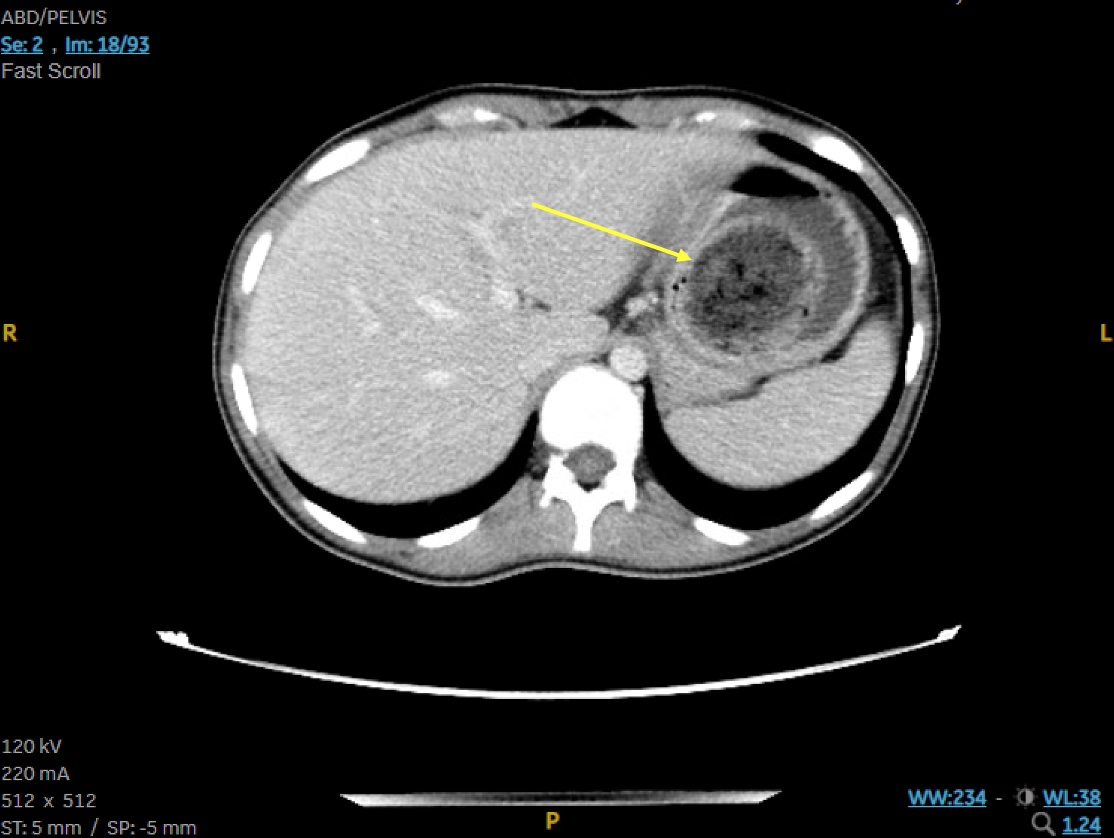

Supplement: Supplementary file 1 [file 11-2-V41-Supp1.jpg]

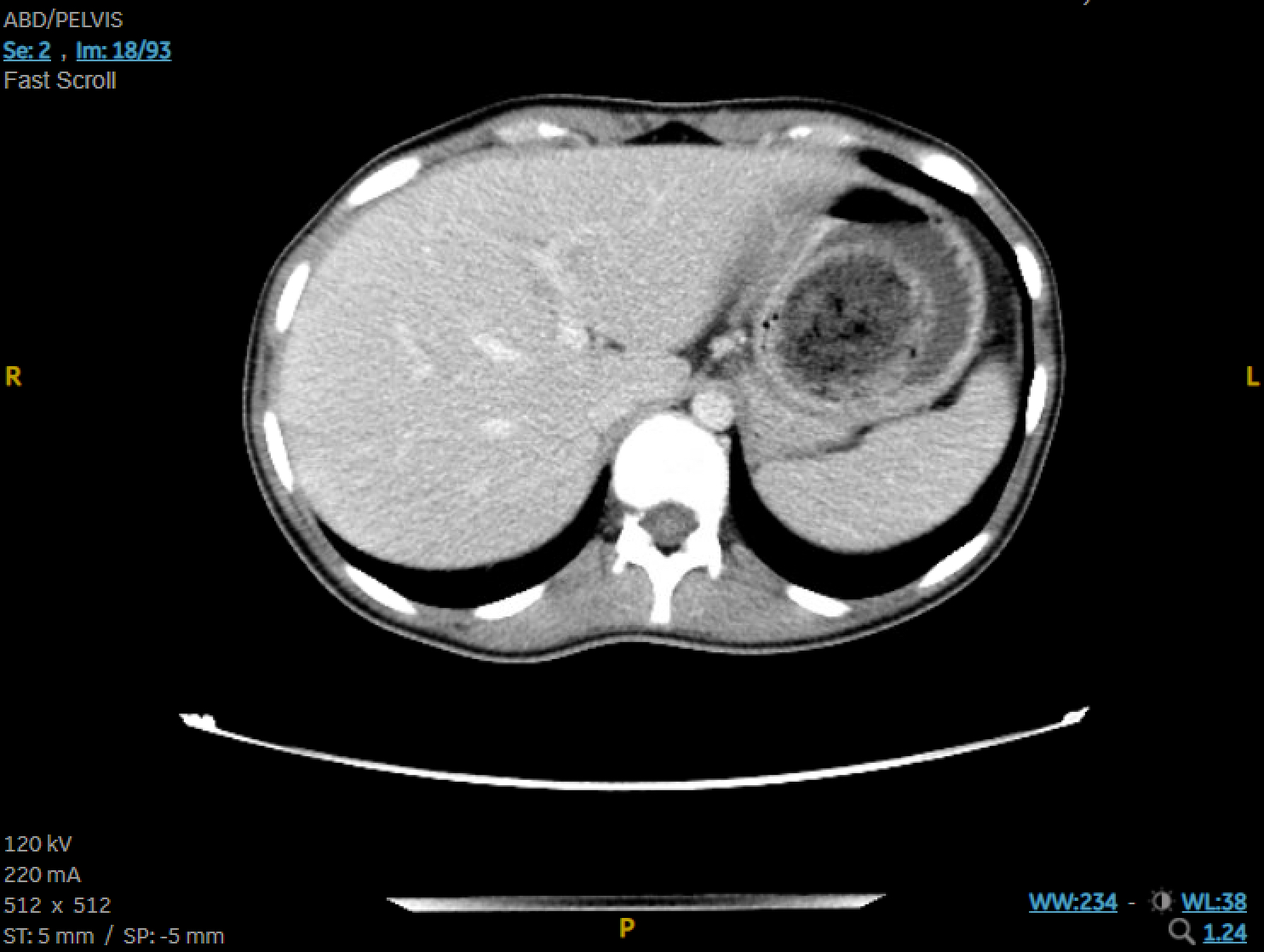

Supplement: Supplementary file 2 [file 11-2-V41-Supp2.jpg]

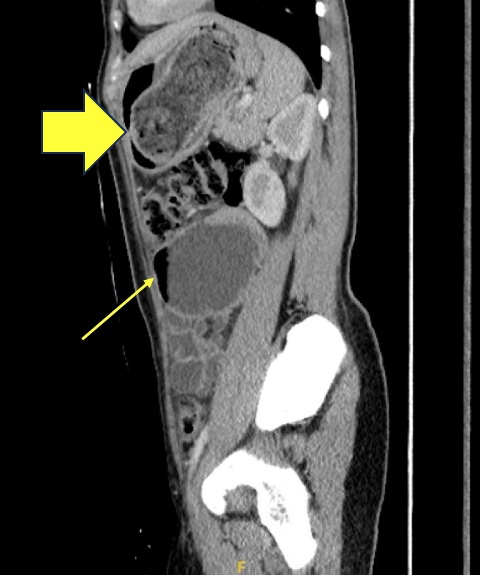

Supplement: Supplementary file 3 [file 11-2-V41-Supp3.jpg]

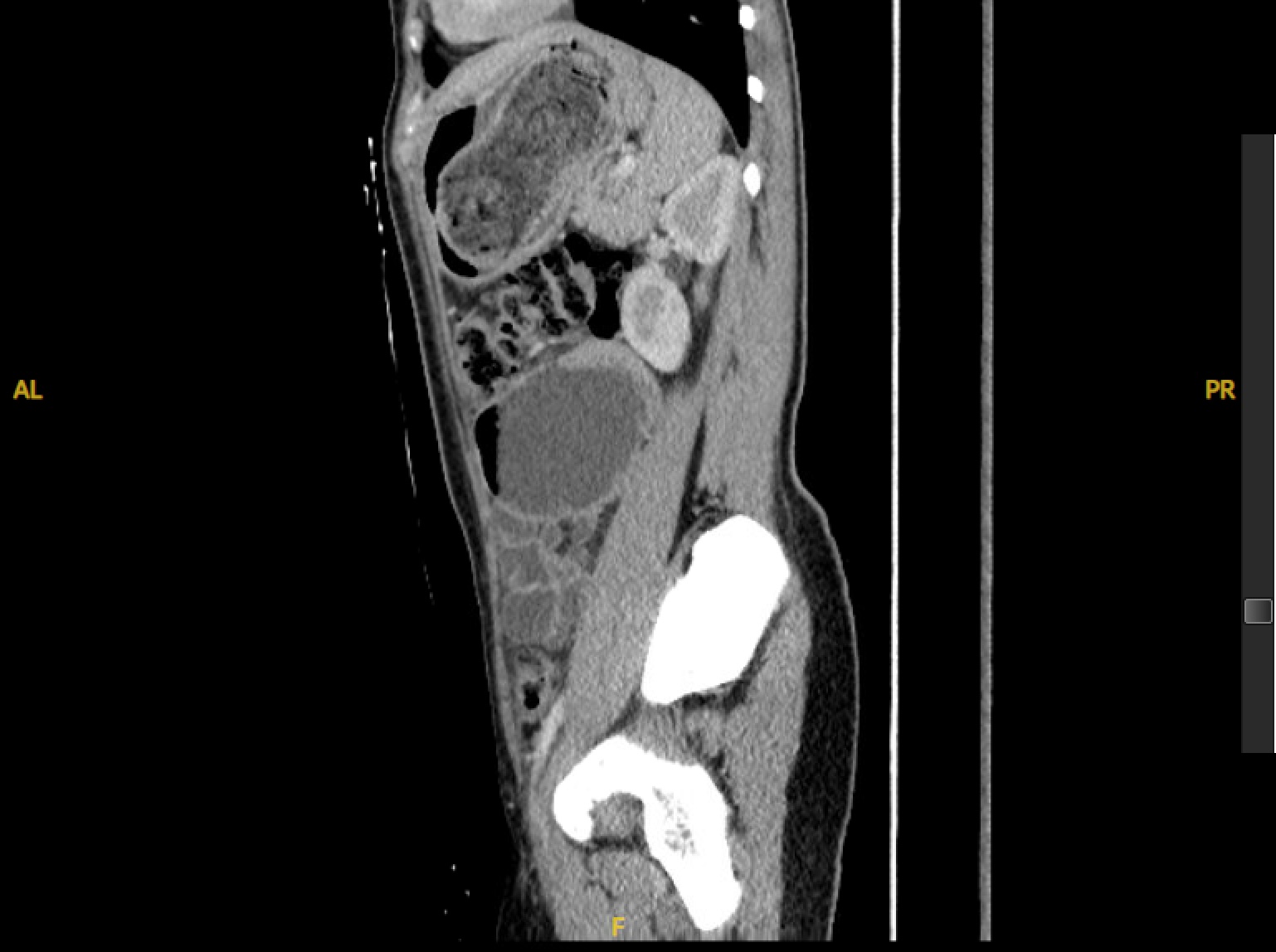

Supplement: Supplementary file 4 [file 11-2-V41-Supp4.jpg]

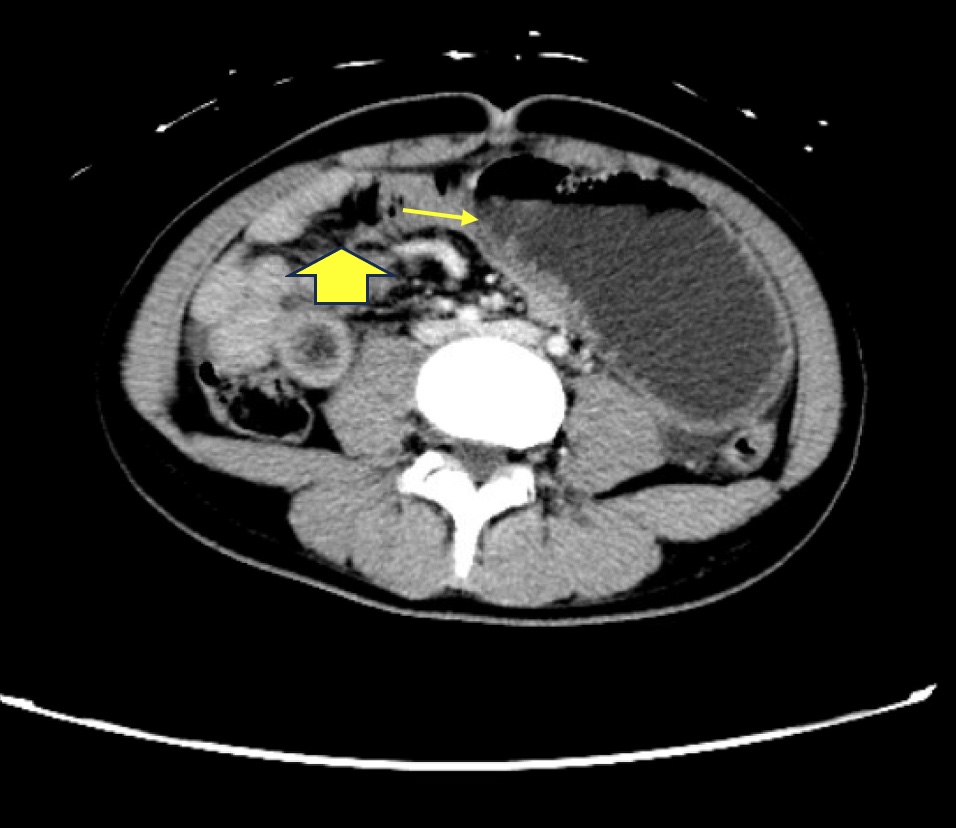

Supplement: Supplementary file 5 [file 11-2-V41-Supp5.jpg]

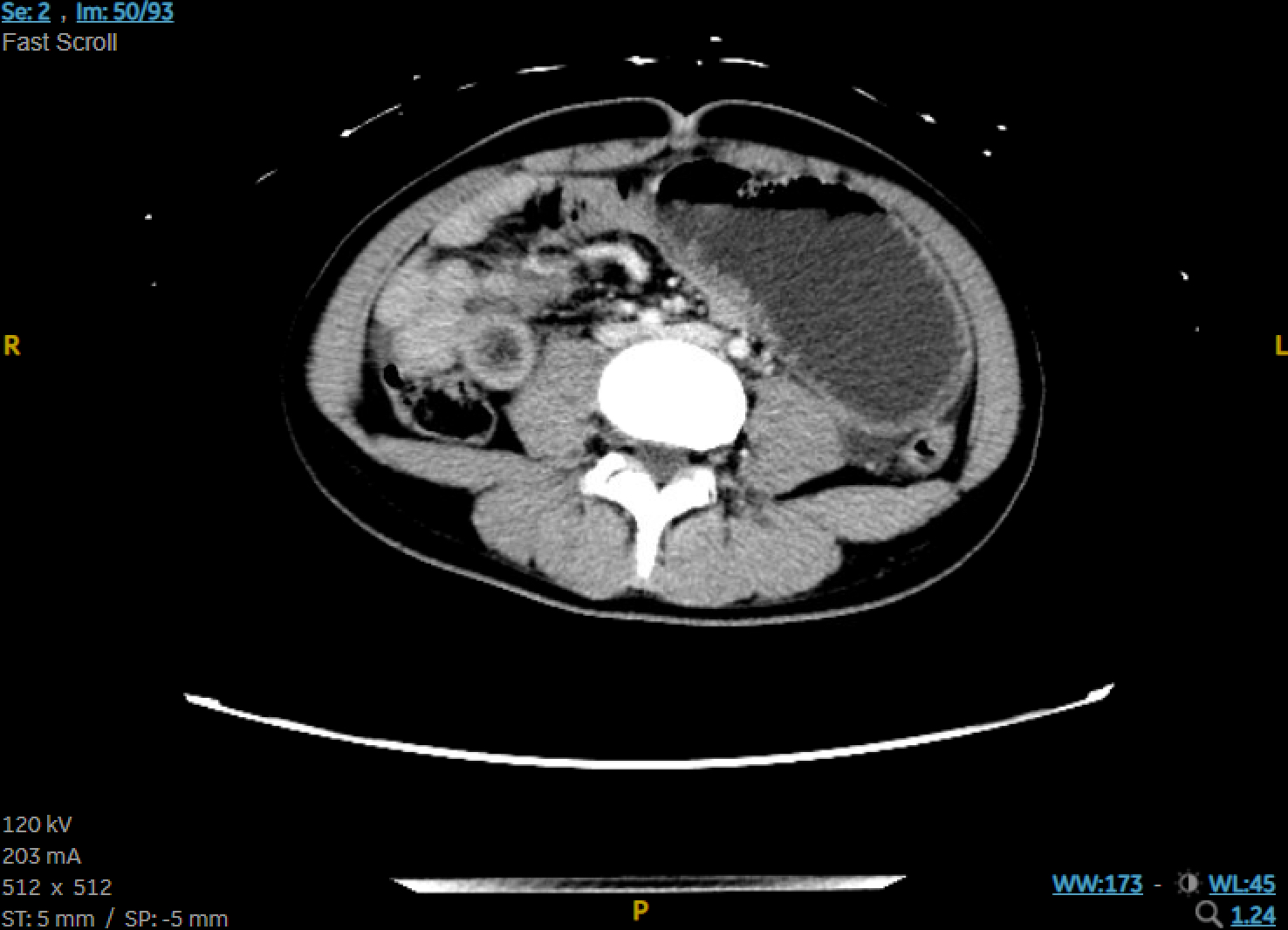

Supplement: Supplementary file 6 [file 11-2-V41-Supp6.jpg]

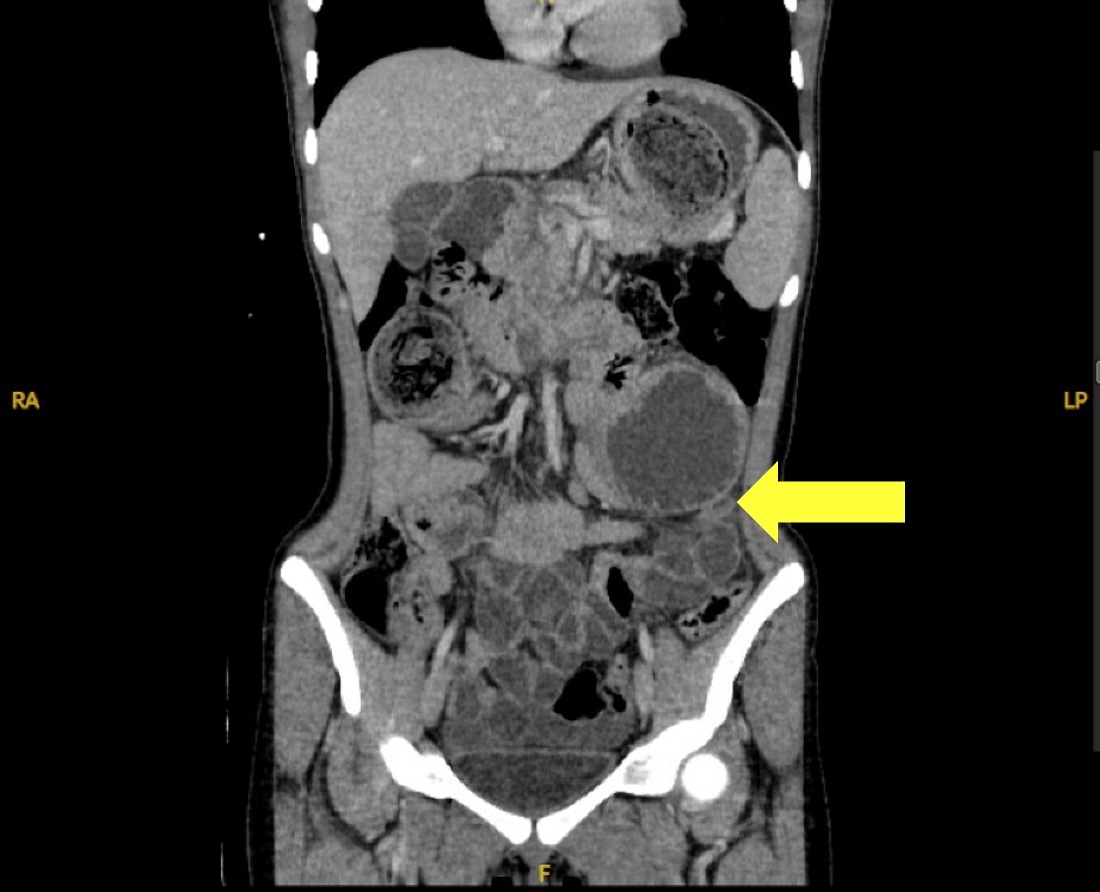

Supplement: Supplementary file 7 [file 11-2-V41-Supp7.jpg]

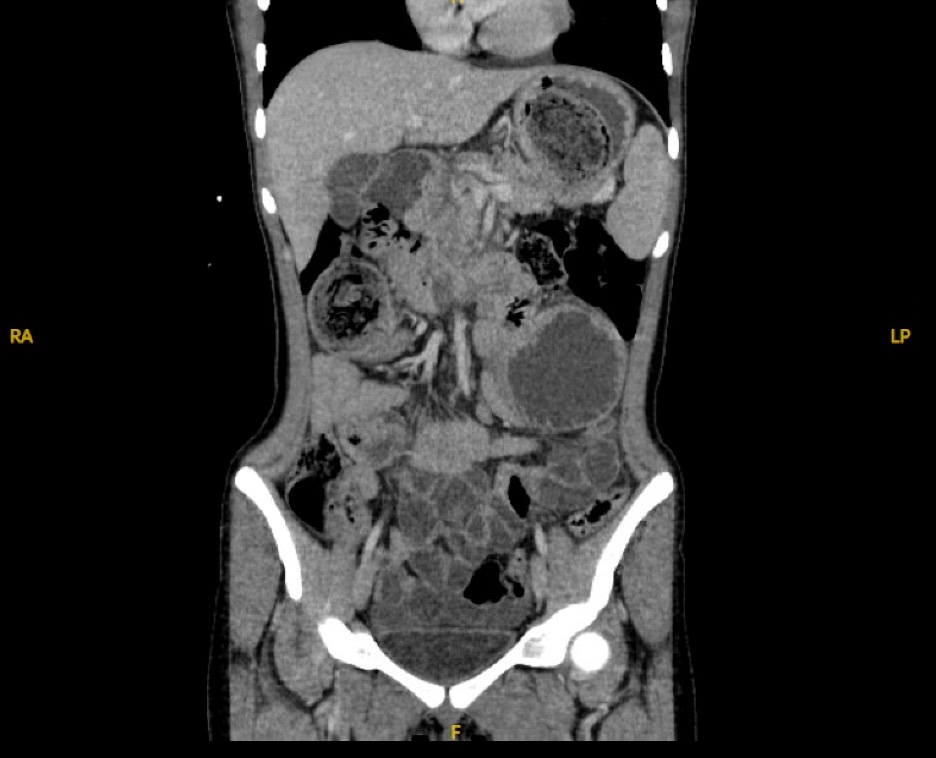

Supplement: Supplementary file 8 [file 11-2-V41-Supp8.jpg]

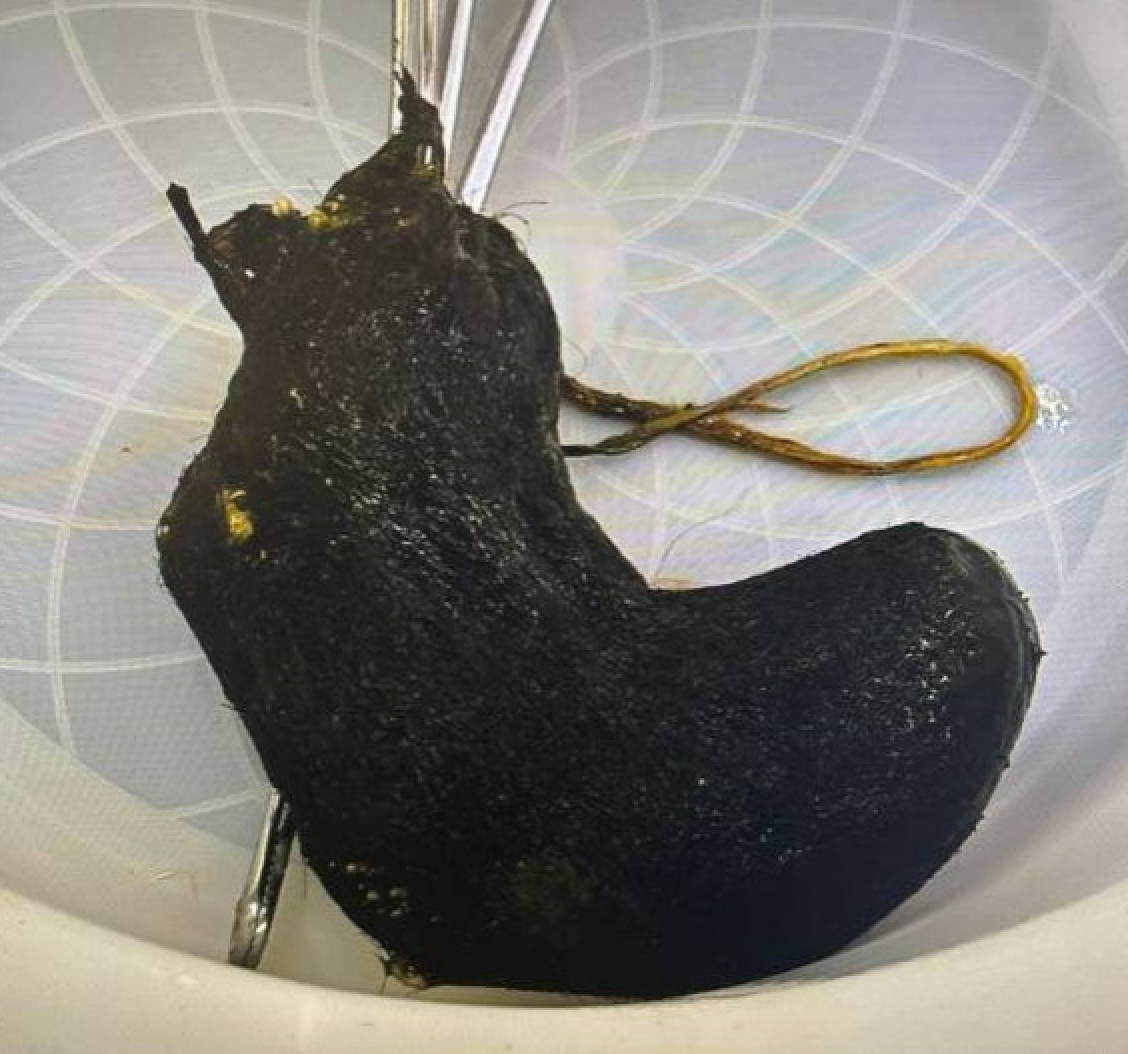

Supplement: Supplementary file 9 [file 11-2-V41-Supp9.jpg]

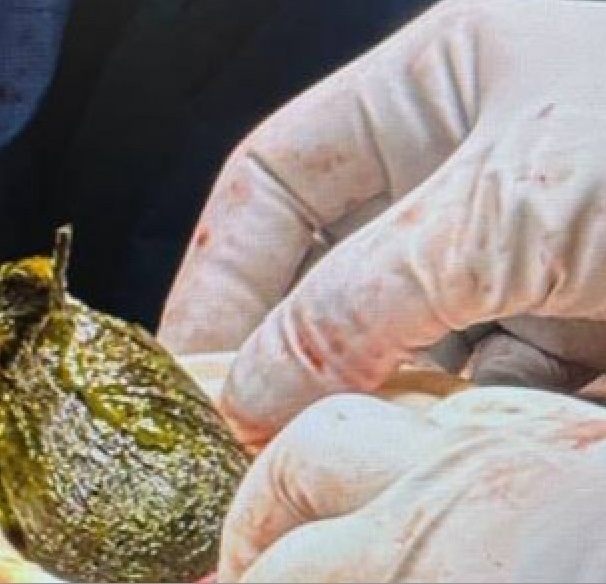

Supplement: Supplementary file 10 [file 11-2-V41-Supp10.jpg]
